# Advancements in the Management of Cervical Intraepithelial Neoplasia: A Comprehensive Review

**DOI:** 10.7759/cureus.58645

**Published:** 2024-04-20

**Authors:** Tejal Waghe, Neema Acharya

**Affiliations:** 1 Obstetrics and Gynaecology, Jawaharlal Nehru Medical College, Datta Meghe Institute of Higher Education and Research, Wardha, IND

**Keywords:** women's health, immunotherapy, treatment modalities, diagnosis, screening, cervical intraepithelial neoplasia (cin)

## Abstract

Cervical intraepithelial neoplasia (CIN) represents a significant precursor to cervical cancer, posing a considerable threat to women's health globally. This comprehensive review examines recent advancements in the management of CIN, encompassing screening, diagnosis, and treatment modalities. The etiology and pathogenesis of CIN are explored alongside an analysis of traditional and emerging screening techniques, including liquid-based cytology and molecular biomarkers. Treatment options, from minimally invasive procedures to immunotherapy approaches, are evaluated for efficacy and potential impact on patient outcomes. Furthermore, this review highlights the implications of these findings for clinical practice, emphasizing the importance of staying abreast of evolving guidelines and integrating innovative strategies into routine care. Recommendations for future research and practice are provided, emphasizing personalized approaches, disparities in access to care, and the exploration of novel therapeutic avenues. By addressing these challenges and opportunities, this review aims to contribute to the ongoing efforts to mitigate the burden of CIN and cervical cancer, ultimately improving women's health outcomes worldwide.

## Introduction and background

Cervical intraepithelial neoplasia (CIN) refers to the abnormal growth of cells on the surface of the cervix, which is the lower part of the uterus that connects to the vagina [1]. CIN is classified based on the extent of cellular abnormalities and is often graded as CIN 1, CIN 2, or CIN 3, with CIN 3 representing the most severe changes. These abnormalities are typically detected through cervical screening tests such as Pap smears and human papillomavirus (HPV) testing [2]. CIN is of significant concern in women's health due to its potential to progress to cervical cancer if left untreated. While not all cases of CIN develop into cancer, early detection and appropriate management are crucial for preventing the progression of pre-cancerous lesions to invasive cervical cancer. Cervical cancer is a leading cause of cancer-related mortality among women globally, underscoring the importance of effective strategies for CIN management [3].

The purpose of this review is to provide a comprehensive overview of advancements in the management of CIN. By examining the latest research and developments in screening, diagnosis, and treatment modalities, this review aims to highlight emerging strategies for detecting and effectively managing CIN. Furthermore, this review seeks to identify remaining challenges and future directions in the field, ultimately contributing to improved outcomes and reduced morbidity associated with CIN and cervical cancer.

## Review

Etiology and pathogenesis of CIN

Risk factors for CIN Development

**Figure 1:**
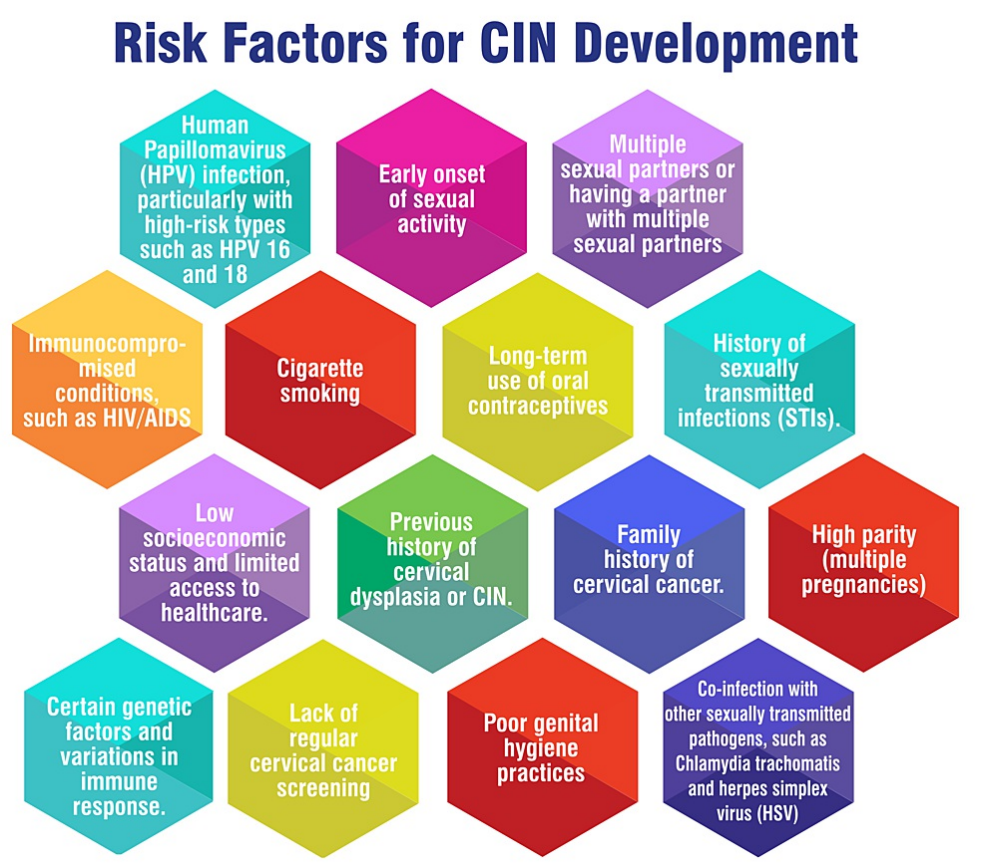
Risk factors for cervical intraepithelial neoplasia (CIN) development Image Credit: Author Tejal Waghe

Risk factors contributing to the development of CIN encompass a variety of elements. Notably, HPV positivity stands out as a significant risk factor for CIN, particularly high-risk HPV types such as HPV 16 and 18, which play a pivotal role [1,4,5]. Furthermore, factors such as the choice of contraceptive method, presence of vaginal infection, smoking habits, and obesity have been identified as additional risk factors that elevate the likelihood of CIN development [1,4]. Research has underscored the association between HPV infection and CIN development, underscoring the critical importance of early detection and management to thwart the progression of cervical cancer [5,6]. Moreover, other risk factors like a failure to wash the vulva after sexual intercourse, low dietary folate intake, and specific occupational backgrounds have also been correlated with an increased risk of CIN [6,7]. Understanding these risk factors is imperative for the implementation of effective screening programs, timely interventions, and personalized management strategies aimed at alleviating the impact of CIN and diminishing the burden of cervical cancer. Figure 1 shows risk factors for CIN development.

Molecular Mechanisms Underlying CIN Progression

The molecular mechanisms underlying CIN progression are intricate processes that profoundly impact tumor initiation, progression, and metastasis. CIN, a common cancer feature, drives tumor evolution and heterogeneity. Studies have elucidated that cancer cells can tolerate certain levels of CIN, granting them adaptive advantages, genetic diversity, and resilience against environmental stresses, including anti-cancer therapies [8]. However, excessive CIN levels can be detrimental to tumor survival and progression, indicating the existence of CIN thresholds that tumor cells can withstand [8]. The research underscores that optimal levels of CIN may enable clonal populations to circumvent selection barriers, highlighting the intricate interplay between CIN levels and tumor development. While low levels of CIN can foster genetic diversity and adaptive advantages, heightened rates of chromosome missegregation can exert tumor-suppressive effects. This dual role of CIN and aneuploidy as both oncogenic triggers and tumor suppressors underscores the complex balance in cancer progression [8]. Moreover, studies have demonstrated that CIN can profoundly affect the tumor microenvironment, modulate immune responses, and influence metabolic signaling pathways. Targeting mechanisms associated with CIN-driven cancer necessitates strategies to counteract the altered immune landscape, exploit metabolic vulnerabilities, manipulate CIN effects, target the extracellular matrix, and address metastasis and STING signaling pathways. Understanding these molecular mechanisms is paramount for developing effective therapeutic interventions to manage CIN-driven cancers and enhance patient outcomes [9].

Role of HPV in CIN Pathogenesis

The role of HPV in the pathogenesis of CIN is pivotal, with high-risk HPV types, especially HPV 16 and 18, strongly associated with the development of this precancerous condition [5]. HPV infection serves as a crucial initiator of CIN, although not all individuals infected with HPV progress to cervical cancer. The infection, particularly by high-risk HPV types, triggers the transformation of cervical epithelial cells, laying the groundwork for CIN development [10,11]. HPV exerts its oncogenic effects primarily through viral oncogenes, notably the E6 and E7 proteins, which disrupt normal cellular processes and promote abnormal cell growth and division in the cervix [10,11]. The presence of HPV infection, particularly high-risk types, is pivotal in CIN pathogenesis, culminating in the formation of precursor lesions that can advance to higher grades of CIN and potentially invasive cervical cancer if left untreated [10,11]. Overall, HPV infection, particularly with high-risk types like HPV 16 and 18, plays a central role in initiating cellular changes in the cervix that can progress to precancerous lesions and, in some instances, invasive cervical cancer [10,11].

Screening and diagnosis of CIN

Current Screening Guidelines

The current screening guidelines for cervical cancer encompass a range of options tailored to different age groups and risk profiles. For individuals aged 21 to 29 years, cytology alone every three years is recommended, while for those aged 30 to 65, the options include HPV testing alone every five years, cytology plus HPV testing every five years, or cytology alone every three years [12,13]. Co-testing with a combination of cytology and HPV testing every five years is another effective screening strategy for individuals in the 30-65 age group [13]. Moreover, the American College of Obstetricians and Gynecologists (ACOG) emphasizes the importance of managing abnormal screening results by the current American Society for Colposcopy and Cervical Pathology (ASCCP) guidelines [13]. It is crucial to follow up promptly with healthcare providers to determine the appropriate course of action based on individual test results. These guidelines enable the detection of CIN effectively while minimizing unnecessary procedures and treatments for false-positive results [13]. The current screening guidelines for cervical cancer recommend tailored approaches based on age and risk factors, highlighting the significance of regular screenings to detect precancerous changes early and prevent the development of invasive cervical cancer.

Screening Modalities: Pap Smear, HPV Testing, Colposcopy

Screening for cervical cancer involves several methods tailored to detect precancerous changes and high-risk HPV types, which are crucial for early intervention and prevention. The Pap smear, or Pap test, is a primary screening tool that collects cervical cells to identify abnormal changes indicative of potential cervical cancer development. Recommended every three years for individuals aged 21 to 29, this test can be extended to every five years when combined with HPV testing for those aged 30 to 65 [14,15]. HPV testing is another pivotal screening method aimed at detecting high-risk HPV types associated with cervical cancer. Often used alongside the Pap test, HPV testing offers a comprehensive assessment of cervical health. Primary HPV screening, particularly for women aged 30 and older, provides a sensitive approach to detecting high-grade cervical dysplasia, a precursor to cervical cancer [14-16].

In cases of abnormal Pap test results or positive HPV tests, colposcopy is performed to evaluate the cervix further. This procedure involves using a colposcope to examine the cervix for any suspicious areas closely. If abnormalities are identified during colposcopy, a biopsy may be conducted to confirm the presence of pre-cancerous or cancerous cells [15,17]. These screening modalities, whether used in combination or as standalone tests, serve as indispensable tools in the early detection and prevention of cervical cancer. Adhering to screening guidelines based on age and risk factors is crucial, as regular screenings significantly reduce the incidence of advanced cervical cancer cases and contribute to improved patient outcomes.

Diagnostic Criteria for CIN Grading

The diagnostic criteria for grading CIN entail thoroughly evaluating cellular changes in cervical tissues. CIN is categorized on a scale from 1 to 3, with CIN 1 denoting mild dysplasia, CIN 2 indicating moderate dysplasia, and CIN 3 representing severe dysplasia or carcinoma in situ [12]. This grading system offers a standardized method for characterizing abnormal epithelial cells and determining the extent of dysplasia, informing clinical management decisions. Histopathological examination plays a pivotal role in both diagnosing and grading CIN, enabling the identification of abnormal cellular features indicative of dysplasia severity. Techniques such as the Pap smear, otherwise known as the Papanicolaou smear, are employed for cytological assessment, evaluating individual cell changes, and assigning CIN grades based on nuclear and cytoplasmic alterations [18]. Parameters, including the nuclear-cytoplasmic ratio, nuclear enlargement, hyperchromasia, irregular chromatin distribution, and the presence of mitotic figures, are among the critical factors assessed to ascertain the grade of CIN [18]. Furthermore, categorizing CIN grades entails a correlation between cytological and histological findings, with CIN 1 corresponding to the low-grade squamous intraepithelial lesion (LSIL). At the same time, CIN 2/3 indicates high-grade squamous intraepithelial lesion (HSIL) [19]. To ensure consistency in the reporting and interpretation of cervical biopsy results, the College of American Pathologists and the ASCCP have established guidelines to standardize the terminology and classification of CIN grades [19].

Traditional treatment approaches for CIN

Overview of Conventional Treatments (Cryotherapy, Loop Electrosurgical Excision Procedure (LEEP), Cone Biopsy)

Conventional treatments for CIN encompass a variety of methods, including cryotherapy, LEEP, and cone biopsy. Cryotherapy, also known as cryocautery, involves freezing abnormal tissue to destroy it effectively and has been a historically popular method for treating CIN [20]. On the other hand, LEEP, a form of electrosurgical excision, removes abnormal tissue using a thin wire loop electrode. This procedure enables precise removal of the affected area and is commonly employed in managing CIN [20]. Additionally, cone biopsy, which may involve cold-knife excision or conization, is a surgical approach to remove a cone-shaped piece of tissue from the cervix for further examination and treatment [21]. These conventional treatments aim to eradicate abnormal cervical tissue effectively while preserving the overall health and function of the cervix. Each method presents its own set of advantages and considerations, with the choice of treatment dependent on factors such as the extent of the lesion, patient characteristics, and the objective of eradicating the abnormal tissue to prevent progression to cervical cancer [20].

Effectiveness and Limitations of Traditional Treatments

Traditional CIN treatments have differing effectiveness and limitations depending on the method employed. Cryotherapy, a widely used destructive technique, has shown cure rates for CIN3 ranging from 77% to 93% in early studies, with some variability in success rates [20]. However, a Cochrane meta-analysis review indicated a lower treatment success rate for CIN3 with cryotherapy than with thermal coagulation, underscoring the importance of selecting the most appropriate method based on the individual case [20,22]. Thermal coagulation, also known as cold coagulation, achieves temperatures of 100-120°C and has emerged as an alternative to cryosurgery, particularly in resource-limited settings [20]. While thermal coagulation presents a viable treatment option, the decision between cryotherapy and thermal coagulation should consider factors such as success rates and the risk of residual disease to ensure optimal patient outcomes [20,22]. Traditional treatments like cryotherapy and thermal coagulation have demonstrated effectiveness in managing CIN. However, they also possess limitations, such as variable success rates and the necessity for careful consideration of the specific characteristics of each case to achieve optimal results [20,22].

Complications Associated with Traditional Treatments

Traditional CIN treatments can yield effectiveness but may also present certain complications. Complications linked with these treatments encompass the risk of harming normal tissue during excisional methods like large loop excision of the transformation zone (LLETZ)/LEEP and cold knife conization, potentially leading to adverse effects impacting future fertility aspirations and also causing obstetric complications [20,23]. Furthermore, destructive methods such as cryotherapy and thermal coagulation may carry the risk of incomplete eradication of abnormal tissue, necessitating vigilant monitoring and follow-up to ensure treatment success [20,23]. Additionally, the treatment decision-making process should consider various patient-specific factors such as age, parity, HPV status, and prior treatments to mitigate the risk of complications and ensure optimal outcomes [20]. Long-term follow-up post-treatment is imperative to identify any residual disease or recurrence, particularly in women aged 50 years or older who are at heightened risk of persistent or recurrent CIN [20]. Adequate follow-up strategies, including colposcopy, cytology, and HPV testing, are vital to effectively monitor patients and avert potential complications or disease progression [20]. While traditional treatments for CIN aim to eradicate abnormal tissue and reduce the risk of cervical cancer, it is essential to acknowledge the potential complications associated with these methods. Close monitoring, appropriate follow-up, and personalized treatment approaches are essential for minimizing risks and ensuring successful outcomes for patients undergoing treatment for CIN.

Advancements in CIN management

Emerging Technologies for CIN Detection and Diagnosis

Liquid-based cytology (LBC): LBC is used in cytopathology, particularly cervical cytology, to prepare samples for examination. Unlike conventional smear tests, where the sample is directly transferred to a microscope slide, LBC involves depositing the sample into a small bottle of preservative liquid. This liquid is then processed to eliminate elements like mucus before a layer of cells is placed on a slide for examination [24]. The origins of LBC can be traced back to efforts to enhance the sensitivity and specificity of the Pap smear. The development of automated screening machines aimed to create standardized slides containing a monolayer of well-stained and well-preserved cells, leading to the evolution of liquid-based gynecologic specimen collection. Liquid-based preparations are favored for their improved fixation, decreased obscuring factors, and standardized cell transfer, all of which contribute to enhanced diagnostic accuracy compared to conventional smears [24]. Two widely used LBC systems approved by the FDA for cervicovaginal testing are SurePath and ThinPrep. In the SurePath method, the sample undergoes vortexing, straining, density gradient layering, and centrifugation, while the ThinPrep method involves special polycarbonate filters and vacuum-assisted cell transfer onto a slide. Liquid-based cytology offers advantages such as improved specimen adequacy, selective loss of extracellular material, and reduced smearing artifacts, enhancing the diagnostic process in cervical screening [25]. The transition from conventional smears to liquid-based cytology has been observed in cervical screening programs, with the United Kingdom adopting LBC in 2008. While early trials indicated increased detection of high-grade CIN (CIN2 and CIN3) with LBC, subsequent studies have not consistently demonstrated a significant difference in detecting these lesions compared to conventional smears [26].

Molecular biomarkers: SCC-A is a well-examined biomarker prominently expressed in various squamous cell cancers, including cervical cancer, offering a potential diagnostic tool for cervical squamous cell carcinoma [27]. Conversely, serum YKL-40 has emerged as a marker specific to cervical adenocarcinoma, presenting a diagnostic avenue tailored to this particular subtype of cervical cancer [27]. Moreover, CTHRC1 has surfaced as a novel biomarker for diagnosing cervical squamous cell carcinoma, showcasing promise for early detection and management strategies [27]. Circulating microRNAs (miRNAs) have exhibited potential as non-invasive biomarkers for identifying cervical lesions, facilitating early diagnosis and risk assessment [27]. Exosomal miRNAs like miR-125a-5p, let-7d-3p, and miR-30d-5p have been recognized as diagnostic biomarkers for cervical cancer and its precursors, highlighting their utility in non-invasive screening modalities [28]. Specific long non-coding RNAs have also been spotlighted as biomarkers for predicting cervical cancer, furnishing insights into disease progression, and identifying potential therapeutic targets [28]. These molecular biomarkers furnish clinicians with valuable insights into diagnosing cervical cancer, prognosticating, and guiding treatment decisions. Their identification and validation propel the advancement of precision medicine in cervical cancer management, aiming to enhance patient outcomes and alleviate the global burden of this disease [27,29,30].

Artificial Intelligence (AI) in CIN diagnosis: AI is revolutionizing the diagnosis of CIN with the development and validation of AI-based analysis software, such as Cervi CARE AI, showcasing promising results in accurately identifying cervical precancerous lesions. This technology can significantly enhance diagnostic accuracy, particularly in colposcopy clinics, by consistently grading impressions and guiding biopsy decisions [31]. Studies have reported varying levels of accuracy for AI in screening CIN 1-3 and adenocarcinoma in situ, ranging from 67% to 98.27%. AI-assisted methods have demonstrated high sensitivity and specificity in clinical cervical cytological screening, offering a valuable tool for triage and improving diagnostic capabilities [32]. AI technology is increasingly utilized in colposcopy and cytological examinations to aid in diagnosing and staging cervical cancer. AI algorithms have proven adept at distinguishing between normal and cancerous Pap smears with remarkable accuracy, providing a standardized and less subjective approach to diagnosis [33]. Researchers have developed AI-based tools for colposcopy examinations that accurately identify CIN lesions and guide tissue sampling locations. These tools exhibit promising results in detecting severe lesions in CIN3 cases and predicting abnormal colposcopy findings in CIN1 and CIN2 cases with high sensitivity and specificity [34]. Integrating AI in CIN diagnosis holds great potential to improve the specificity and accuracy of screening programs, reduce misdiagnosis rates, and enhance diagnostic performance in cervical cancer care. Ongoing research endeavors aim to refine AI algorithms further, explore chronological changes in abnormal colposcopy findings, and enhance the predictive capabilities of AI in histopathologic diagnosis [34].

Minimally Invasive Treatment Modalities

Thermal ablation techniques: Minimally invasive treatment modalities, particularly thermal ablation techniques, are pivotal in managing various tumors. Thermal ablation involves applying thermal energy to heat or cool tissue to cytotoxic levels, typically less than -40°C or more than 60°C, to induce tumor destruction [35,36]. Several modalities are utilized in thermal ablation, including radiofrequency, microwave, laser, high-intensity focused ultrasound, cryoablation, and irreversible electroporation, each tailored to specific indications and optimal uses [35]. Radiofrequency ablation (RFA) employs high-frequency alternating currents to cause tissue damage, microwave ablation (MWA) generates heat through electromagnetic waves, and cryoablation rapidly cools tissue to induce cell death [36]. Laser ablation (LA) is another hyperthermic technique that utilizes light energy to target tissues [36]. These modalities offer minimally invasive approaches for patients who may not be suitable for surgery or have failed other treatments, such as chemotherapy or radiotherapy. In the context of non-small cell lung cancer (NSCLC), thermal ablation techniques like RFA, MWA, and cryoablation have demonstrated promise in providing alternative curative-intent therapy for medically inoperable patients with Stage I NSCLC [36]. These techniques have been compared to stereotactic ablative body radiotherapy (SABR) and have shown comparable overall survival benefits and progression-free survival rates [36]. Thermal ablation techniques, as minimally invasive treatment modalities, offer a valuable option for managing tumors in various organs, including the liver, kidney, bone, lung, and particularly in the context of NSCLC. These techniques effectively destroy tumors while minimizing the invasiveness associated with traditional surgical procedures, thereby underscoring their significance in modern oncology practices [35,36].

Laser therapy: Recent research has emphasized minimally invasive treatment modalities, particularly laser therapy. Laser therapy, harnessing focused light, presents a precise and less invasive approach to addressing various medical conditions. This technique enables high precision in surgical procedures, reducing pain, swelling, and scarring compared to traditional surgical methods [37]. The applications of laser therapy are diverse, ranging from tumor removal and cancer treatment to kidney stone removal, prostate procedures, detached retina repair, vision enhancement, hair loss treatment, pain management, and cosmetic procedures such as scar, tattoo, and wrinkle removal [37]. In the realm of periodontal and peri-implant therapy, lasers like erbium, chromium-doped: yttrium, scandium, gallium, and garnet (Er,Cr:YSGG) have been explored for their efficacy in nonsurgical and minimally invasive surgical interventions. While specific studies have not demonstrated statistically significant differences in pocket reduction and clinical attachment gain with adjunctive laser therapy, successful clinical outcomes have been reported, underscoring the necessity for further evidence-based research [38]. Furthermore, low-level laser therapy (LLLT) has effectively alleviated pain and fostered tissue regeneration in musculoskeletal disorders, rendering it a valuable tool in physiotherapy [39]. Overall, advancements in minimally invasive treatment modalities, particularly laser therapy, present a promising avenue for precise, effective, and less invasive medical interventions across various conditions. This underscores the importance of ongoing research to explore further and optimize these techniques [37,40].

Photodynamic therapy: Minimally invasive treatment modalities, particularly photodynamic therapy (PDT), have emerged as a promising approach for various conditions, including cancer and precancerous lesions. PDT employs photosensitizer drugs activated by specific wavelengths of light to induce cell death in targeted tissues [41]. This treatment modality has demonstrated effectiveness in addressing diseases such as skin cancer, lung cancer, esophageal cancer, and actinic keratosis, among others [42]. The mechanism of action in PDT involves the generation of reactive oxygen species (ROS) when cells containing photosensitizers are exposed to light, resulting in direct cytotoxic effects on tumor cells, vascular damage to tumors, and the initiation of an inflammatory reaction that may prompt the immune system to target tumor cells [43]. The success of PDT hinges on factors such as the type and dosage of photosensitizer utilized, the drug-to-light interval, light dosage, and fluence rate [44]. PDT offers several advantages over traditional treatments, including minimal scarring, no known long-term side effects, outpatient procedures, and precise antitumor activity compared to interventions like surgery [44]. Its capacity to target local diseased cells and tissues makes it a preferred choice for conditions such as oral lichen planus and precancerous lesions in the head and neck region [45].

Immunotherapy Approaches for CIN treatment

HPV vaccination: Immunotherapy approaches for CIN treatment focusing on HPV vaccination have demonstrated promising results in recent research. Immunotherapy, encompassing checkpoint blockade therapy and other innovative strategies, is emerging as a valuable treatment option for cervical cancer patients [46]. Notably, pembrolizumab, an anti-PD-1 monoclonal antibody, has garnered FDA approval for cervical cancer treatment, although its response rate remains relatively modest [46]. Immune checkpoint inhibitors, such as pembrolizumab, target proteins like PD-1 to bolster the immune response against cancer cells, potentially leading to tumor reduction or growth deceleration [47]. Furthermore, novel immune checkpoints like CTLA-4, TIGIT, LAG-3, TIM-3, and A2AR have surfaced, providing novel avenues for enhancing antitumor efficacy in cervical cancer treatment [46]. Combining PD-1/PD-L1 inhibitors with various immunotherapies or biotherapies has shown promise in augmenting treatment outcomes [46]. Moreover, innovative approaches such as DNA vaccines, RNA-based vaccines, peptide- and protein-based vaccines, and engineered T-cell therapy are under exploration to amplify the immune system's response against HPV-associated cervical cancer [48]. These strategies target specific antigens implicated in cervical cancer, potentially yielding improved treatment responses and enhanced patient outcomes. The evolving landscape of immunotherapy for CIN treatment, mainly through HPV vaccination and other innovative strategies, holds significant promise in enhancing the management and outcomes of cervical cancer patients. Continuous research efforts and advancements in immunotherapy are paving the way for more effective and tailored treatment options in combatting cervical cancer [46,48].

**Figure 2:**
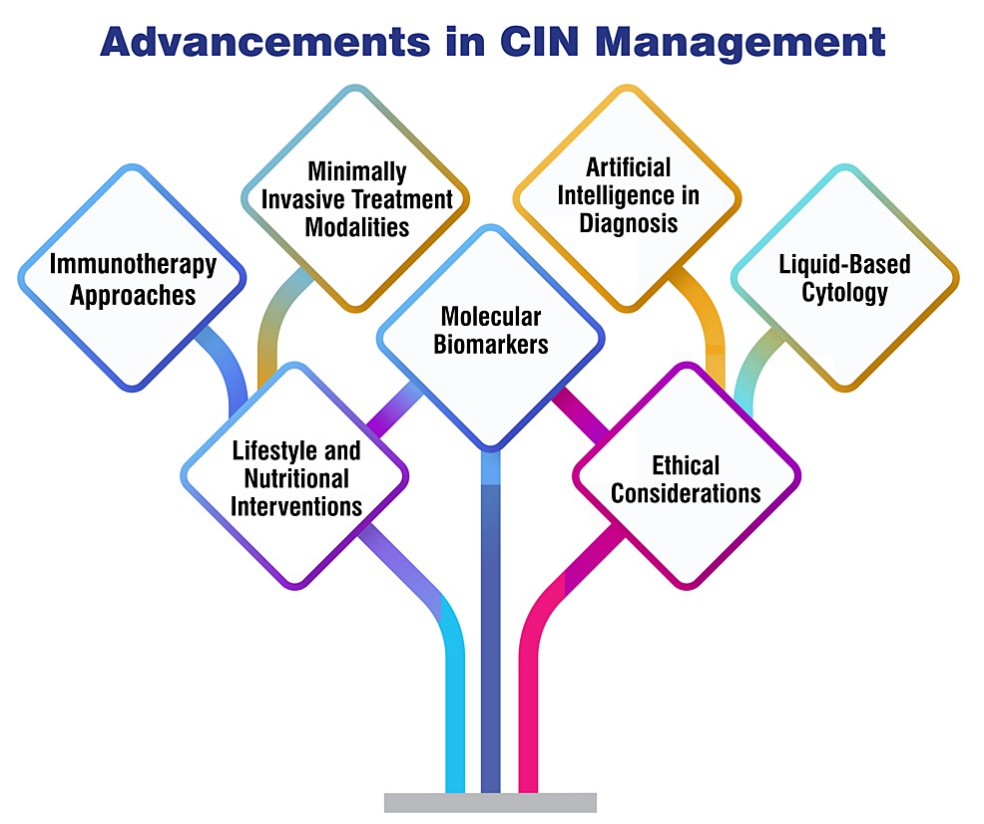
Advancements in cervical intraepithelial neoplasia (CIN) management Image Credit: Author Tejal Waghe

Therapeutic vaccines: Immunotherapy approaches for treating CIN have exhibited promising outcomes in recent research endeavors. A variety of therapeutic vaccines have been developed, including peptide/protein-based vaccines, nucleic acid-based vaccines (DNA), and live vector-based vaccines (bacterial or viral), all designed to target the E6 and E7 proteins associated with HPV infection [48-51]. These vaccines are intended to stimulate specific immune responses against HPV antigens, aiming to counteract the progression of CIN. Clinical trials have demonstrated the tolerability and immunogenicity of these vaccines, with some exhibiting notable immune responses such as E7-specific cytotoxic T lymphocyte (CTL) responses and HPV-specific IFNy-associated T-cell responses [48-51]. Reported efficacy rates for these vaccines in regressing CIN 2/3 lesions to either no CIN or CIN1 range from 17% to 59% [51]. Moreover, ongoing trials are investigating combination regimens, such as the GX-188E therapeutic DNA vaccine combined with pembrolizumab, which has shown promise in treating recurrent cervical cancer [46]. Additionally, the utilization of immune checkpoint inhibitors, like pembrolizumab, has emerged as a targeted therapy for cervical cancer. These inhibitors block PD-1 to augment the immune response against cancer cells, resulting in tumor shrinkage or growth inhibition [47]. They can be administered alone or in conjunction with chemotherapy for advanced or recurrent cases of cervical cancer. Immunotherapy approaches, encompassing therapeutic vaccines and immune checkpoint inhibitors, present novel pathways for managing CIN, showing potential in bolstering immune responses against HPV antigens and enhancing treatment outcomes for individuals with cervical neoplasia [52]. Figure 2 shows advancements in CIN management.

Role of Nutritional and Lifestyle Interventions in CIN Management

Nutritional and lifestyle interventions are pivotal in managing CIN. Research indicates that dietary factors can influence CIN risk, with specific nutrients such as carotenoids, folate, vitamins C and E, and other B vitamins potentially affecting CIN development and progression [53,54]. Studies have demonstrated that a low intake of fruits and vegetables correlates with an elevated risk of CIN. In contrast, higher serum levels of specific nutrients like carotenoids and gamma-tocopherol are associated with a decreased risk of high-grade CIN [53,54]. Additionally, lifestyle modifications encompassing attention to diet, social habits, sexual behavior, and vaccination can significantly contribute to cervical cancer prevention and CIN management [53]. Factors such as alcohol consumption and smoking have been linked to increased risks, while maintaining a diet abundant in fruits, vegetables, and nutrients like zinc, iron, niacin, potassium, and phosphorus may potentially diminish the risk of cervical cancer and CIN progression [53]. Lifestyle choices, including steering clear of high-risk diets, maintaining a healthy diet, and undergoing HPV vaccination, constitute crucial components of a comprehensive approach to CIN management. The integration of nutritional considerations and lifestyle modifications into CIN management is imperative for mitigating the risk of cervical cancer progression and enhancing patient outcomes [53,54].

Challenges and future directions

Remaining Challenges in CIN Management

As elucidated in recent research, persistent challenges persist in managing CIN. A notable obstacle is the lack of consensus among colposcopists regarding managing CIN 2 lesions, resulting in divergent opinions on regression likelihood and the appropriate selection criteria for conservative management [55]. This disparity in viewpoints poses a significant challenge in clinical practice, underscoring the need for standardized guidelines to streamline decision-making processes. Additionally, the clinical trajectory of untreated CIN 2 remains a subject of interest, with studies indicating high regression rates alongside the potential for progression to more severe stages such as CIN 3 or invasive cancer [1]. Grasping the natural history of CIN 2 and its clinical outcomes is pivotal for guiding clinicians in making well-informed management decisions and optimizing patient care.

Further challenges lie in refining the criteria for selecting women for various management strategies, particularly in cases where observation or conservative management is contemplated [1,55]. This necessitates a more profound comprehension of CIN 2 lesion behavior, factors influencing progression, and the formulation of personalized treatment approaches. The ongoing challenges in CIN management revolve around establishing consensus in clinical practice, augmenting knowledge concerning CIN 2's natural history, fine-tuning selection criteria for management strategies, and devising personalized treatment approaches to ameliorate patient outcomes and mitigate the burden of cervical cancer [1,55]. Addressing these challenges is imperative for advancing the field and ensuring optimal care for individuals grappling with CIN.

Potential Areas for Future Research and Innovation

Exploring innovative approaches with emerging technologies: Research suggests delving into innovative approaches utilizing emerging technologies like digital health interventions, AI, and machine learning (ML) to enhance awareness, screening, and treatment provision for cervical cancer care [56].

Enhancing awareness dissemination and screening facilitation: Future research could address the deficiency in studies related to awareness dissemination, screening facilitation, and treatment provision for cervical cancer. This includes developing sophisticated machine-learning models for screening, incorporating additional features in machine-learning research, and creating more user-friendly applications for cervical cancer care [56].

Optimizing treatment modalities: There is a need to investigate further the impact of treatments across different stages of cervical cancer and explore ways to scale up treatment modalities to improve patient outcomes and increase survival rates [31,56].

Integration of digital interventions: Future research could emphasize the integration of mobile applications, AI, and ML as critical components of digital interventions in cervical cancer care. These technologies offer platforms for health monitoring, education, personalized healthcare services, and real-time health data management, potentially revolutionizing the field [31,56].

Assessment of the feasibility of detection and treatment: Research could focus on detecting suspected cancer and determining eligibility for direct treatment with innovative techniques like thermal ablation. This area holds promise for improving diagnostic accuracy and treatment outcomes in cervical cancer care [57]. Future research and innovation in the management of CIN should aim to leverage emerging technologies, enhance awareness and screening efforts, optimize treatment modalities, integrate digital interventions, and explore novel approaches for detection and treatment to advance patient care and outcomes in the fight against cervical cancer.

Ethical Considerations in Adopting Novel Therapeutic Approaches

Ethical considerations are pivotal in adopting novel therapeutic approaches, particularly in clinical research and treatment. The emergence of innovative treatments such as psychedelics, ketamine, and neuromodulatory technologies introduces novel ethical dilemmas that necessitate careful examination [58]. These advancements bring about challenges related to informed consent, the impact of expectancy on clinical response, and distributive justice, emphasizing the need for a comprehensive ethical framework to guide their implementation [58]. Informed consent is a cornerstone that demands special attention when contemplating swift and innovative treatments in psychiatry. Clinicians and researchers must ensure that individuals thoroughly understand the treatments, their potential benefits, risks, and implications, especially considering the unique attributes of these interventions that may influence patient expectations and outcomes [59]. Additionally, integrating these treatments into clinical practice requires a respectful acknowledgment of cultural backgrounds and the active involvement of diverse communities in the decision-making process [59].

The influence of expectancy on clinical response emerges as another critical ethical consideration. Patient expectations can significantly shape treatment outcomes, particularly in novel therapy scenarios where rapid symptom alleviation may correlate with heightened patient expectancy [60]. Clinicians and researchers must navigate the complexities of managing patient expectations by accurately conveying treatment objectives and implications and integrating these novel therapies into comprehensive, long-term treatment plans [60]. Ethical considerations in adopting novel therapeutic approaches underscore the significance of upholding principles such as informed consent, managing patient expectations, and ensuring equitable access to innovative treatments while prioritizing patient well-being and autonomy [60]. Addressing these ethical challenges thoughtfully enables the field to responsibly and ethically integrate novel therapies into clinical practice.

## Conclusions

In conclusion, this comprehensive review has highlighted significant advancements in managing CIN. Through the examination of various screening techniques, including liquid-based cytology and molecular biomarkers, improved accuracy and sensitivity in CIN detection have been achieved. Moreover, the exploration of minimally invasive treatment modalities such as thermal ablation and laser therapy offers promising alternatives to traditional surgical interventions, potentially reducing morbidity associated with treatment. Additionally, immunotherapy approaches, such as HPV vaccination and immune checkpoint inhibitors, show considerable potential in preventing and treating CIN by targeting underlying viral infections and enhancing the immune response. The review also underscores the importance of educating patients about the benefits of HPV vaccination and adopting healthy lifestyle behaviors to mitigate CIN progression risks. Future research should focus on long-term efficacy and safety assessments of emerging treatments, exploring novel immunotherapy strategies, and addressing disparities in access to screening and treatment services to ensure equitable care for all individuals affected by CIN. By embracing these recommendations, clinicians and researchers can continue to advance the field of CIN management and make significant strides in reducing the global burden of cervical cancer.
